# The red ear

**DOI:** 10.11604/pamj.2018.30.34.15020

**Published:** 2018-05-16

**Authors:** Aryé Weinberg, Ralph Magritz

**Affiliations:** 1 Prosper-Hospital, Department of Oto-rhino-laryngology, Head and Neck Surgery, Recklinghausen, Germany

**Keywords:** Red ear, mastoiditis, insect bite

## Image in medicine

A 5-year-old boy was presented to our out-patient clinic by his father with a painful swollen red left ear (A). The father stated that his son has been suffering from an upper- respiratory tract infection and that his left ear has been hurting since the other day. Hearing loss was denied. Clinical examination showed a painfull swelling of the left ear causing it to stick out. Behind the ear an insect bite was noticed, probably from a mosquito (B). Otoscopy showed a white transparent ear drum with no signs of infection. Thus an acute mastoiditis based on a middle ear infection was excluded. The patient was treated for a mosquito bite with a topical coricoidsteroid gel and cooling. The boy totally recovered after two days.

**Figure 1:**
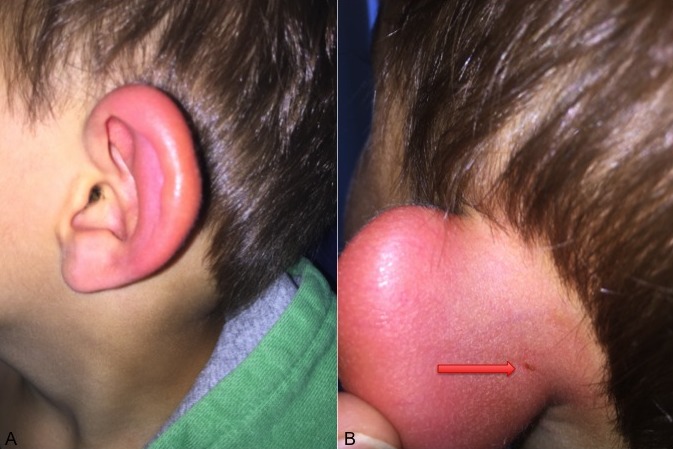
A) swollen red ear; B) ear with mosquito bite

